# Bioavailability of Metal Ions and Evolutionary Adaptation 

**DOI:** 10.3390/life2040274

**Published:** 2012-10-29

**Authors:** Rolando P. Hong Enriquez, Trang N. Do

**Affiliations:** International School for Advanced Studies (SISSA), Via Bonomea 265, 34151 Trieste, Italy; E-Mail: dntrang@sissa.it

**Keywords:** metalloproteins, Fenton reaction, bioavailability, aerobic respiration, photosynthesis, organelle

## Abstract

The evolution of life on earth has been a long process that began nearly 3.5 × 10^9^ years ago. In their initial moments, evolution was mainly influenced by anaerobic environments; with the rise of O_2_ and the corresponding change in bioavailability of metal ions, new mechanisms of survival were created. Here we review the relationships between ancient atmospheric conditions, metal ion bioavailability and adaptation of metals homeostasis during early evolution. A general picture linking geochemistry, biochemistry and homeostasis is supported by the reviewed literature and is further illustrated in this report using simple database searches.

## 1. Introduction

Observing the space we have come to realize how the history of our own planet began. Today it is common knowledge in cosmology how our sun was formed from an explosion of a supernova and how the interstellar dust that did not collapse into the sun formed, in time, the earth and the other planets of our solar system. Our planet was in fact formed 4.578 Ga (billions of years ago) [1]. Within its first 500 million years the earth rapidly differentiated: the heavier Fe and Ni collapsed into the center, creating the core and the magnetic field of our planet, while an early fast “outgassing” created more than 85% of a primitive atmosphere in which N_2_, CO_2_, and H_2_O were probably already present [2]; a slower outgassing process continues even today [3]. Besides the H_2_O coming from outgassing, a smaller portion of water in our planet came from collisions with comets [4]. In a few million years the surface of the earth rapidly cooled down, giving rise to heavy rains that ultimately formed our oceans ∼4.4 Ga ago [5]. The conditions that were created at this time are believed to be sufficient to sustain the origin of life [6]; additionally, some evidence points to a decreased destructive effect of UV radiation in the oceans due to a shielding effect of the oceanic waters on the first organic molecules and microorganisms [7]. This hypothesis is somewhat controversial in aqueous environment due to the poor absorption of UV by the oceans and still does not explain a putative shielding effect over the first photosynthetic organisms that used visible light and lived in dry environments (e.g., stromatolites? [8]). On the other hand, those initial times of our planet were immensely violent, and although the water from the oceans probably brought some protection from meteoritic collisions [9], the repeating impacts of large planetary bodies could in principle caused the extinction of life several times if it existed at all [9]. In fact, the earth debris caused by one of those impacts probably created our moon [10]. The heavy meteoritic collisions ended ∼3.9 Ga ago and they were so intense that there is barely any geological record of the earlier conditions in our planet [11]. Talking about life before this time is therefore a highly speculative exercise. The origin of life in our planet probably passed through synthetic prebiotic processes in which geochemistry slowly evolved into a primitive biochemistry [12]. For several millions of years during these early beginnings there was practically no O_2_ free in out atmosphere. Profound changes would still be needed in our planet until the rise of our modern O_2_ rich atmosphere. The effects of these changes on the bioavailability of relevant metal ions and the subsequent effects for metal ion homeostasis are briefly reviewed and commented in this work. 

## 2. Origin and Evolution of O_2_ on Earth

How our planet became oxygenated is still debated. A usually mentioned cause for this phenomenon is the rise of oxygenic photosynthesis [13], a process that actually started 200 million years before of the O_2_ started rising [14]. Other possible explanations are, for example, the abrupt and permanent decrease of oxygen sinks like submarine vulcanism [15], the change in the composition of volcanic gases [16] or in the degassing pressure of volcanoes [17]. Whatever the timing and real mechanism of these processes, most researchers do agree in indicating the presence of an initial small quantity of O_2_ [18] and aerobic respiration [19] in the early atmosphere. In fact, this initial aerobic respiration was probably instrumental in keeping the relatively low levels of O_2_ preceding the origin of photosystem II [20] or whatever combination of causes that lead to the first large and fast increment of O_2_ ∼2.45 billion years ago and now known as the Great Oxidation Event (GOE) [21]. This first GOE increased only the atmospheric O_2_, leaving the oceans unaffected [22], a situation that lasted ∼1.8 Ga when a second GOE that occurred about 0.6–0.8 Ga ago provoked the oxygenation of deep oceans. The global consequences of the GOEs were substantial; e.g., they have been associated with the first two ice ages [23,24]. However, on the biological point of view, different associations can be made: (i) the first GOE probably caused massive extinction of anaerobic life [25] and an increased O_2_ related detoxification and utilization capacity [26], (ii) the second GOE is generally related to the emergence of multicellular life [22]. 

Given the current importance of O_2_ for the life in our planet, the high toxic potential of O_2_ expressed through metal ion catalyzed reactions and the differential changes in the aggregation states of metal ion redox species resulting from their reactions with O_2_, it is interesting to examine the biological implications of GOEs. In the next section we start our analysis of these phenomena by resuming the effect of increasing quantities of O_2_ on the bioavailability of metal ions, concentrating our attention in two prototypical cases: copper (Cu) and iron (Fe). 

## 3. Rise of O_2_ and Change in Metal Ion Availability

Before the rise of O_2_ in the atmosphere, the primitive oceans were saturated with several metal ions like Fe^2+^, Mn^2+^ or Mo^6+^ among others [27], with Fe^2+^ being the most common among them. The catalytic machinery of early life surely took advantage of these conditions and abundant biochemical evidence of this fact can still be found in bacterial enzymes [28]. On the other hand, the rise of oxygen brought immediate geochemical effects; O_2_ reacted with soluble iron (Fe^2+^) in the waters of ancient seas to form layers of insoluble iron oxides (Fe^3+^), these layered oxides created the mineral now known as banded iron [29]. Independently of the dynamical routes and factors governing the banded iron formation, a final result of this process was certainly a decrease of soluble and bioavailable Fe^2+^ in the oceans. Contrary to the case of Fe, Cu becomes more soluble when reacting with O_2_ and passing from Cu^1+^ to Cu^2+^ [27]. The drastic changes in O_2_ and metal bioavailability prototypically exemplified by Fe^2+^ and Cu^1+^ constrained early life forms to avoid, adapt, detoxify or use in different ways these elements, and the reason is the paradoxical dichotomy between the toxicity and energetic advantages of O_2_ mediated processes. A brief exploration of these topics follows. 

## 4. Adaptation of Metals Homeostasis Caused by the Rise of O_2_

In this section we review a few aspects related to the homeostatic metabolic adaptations resulting from the rise of O_2_ levels. The included topics will cover the physical-chemical basis of O_2_ toxicity, the intracellular handling of labile ion pools, the intracellular metal storage mechanisms, the reduction of O_2_-related toxicity through the control of the oxidative stress, and finally the now ubiqui­tous but still surprising evolutionary mechanisms of O_2_ transport using potentially reactive metal ions. 

The information in this section is occasionally commented with the help of simple database searches. The use of databases in this mini-review is for illustrative purposes and no absolute claim is done based on statistically sophisticated tests. We performed text data-mining in the Uniprot database [30], specifically using the Protein KnowledgeBase (UniprotKB). UniprotKB is non-redundant, as experimentally determined identical sequences coming from different sources are represented as a single entry in the database. Using the integrated online services, we performed advanced boolean searches on several fields (e.g., taxonomy, keywords, enzyme classification, metal binding proteins, *etc.*); all the searches were performed using Uniprot predefined and reviewed fields. We reduced the redundancy of the obtained set of sequences to a final group of canonical (unique) genes per species. 

### 4.1. Basis of O_2_ Toxicity

The toxicity of oxygen has physicochemical basis in its tendency to acquire electrons from other molecules by non-enzymatic autoxidations. Because O_2_ cannot accommodate a spin-matched pair, electrons must be acquired one at a time; the breaking up of electron pairs results in free radical formation. Specifically, the one electron reduction of the ground state molecular oxygen produces the superoxide, *·O*_2_− . A second single reduction produces hydrogen peroxide, H_2_O_2_, which is not formally a free radical but together with other oxygen free radicals constitute the so-called Reactive Oxygen Species (ROS). A relevant example can be found through the Haber–Weiss mechanism [31] of the Fe^2+^ catalyzed Fenton reaction [32]:

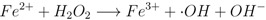
(1)


We can also write a similar equation for Cu^+^:

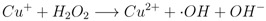
(2)

Much of the toxicity associated with transition metals is caused by Fenton chemistry. Although this process is normally referred to the reactions involving Fe, in reality Cu is more toxic for a variety of reasons. Here we mention a few examples: (i) Cu^2+^ provokes higher oxidative damages than Fe^3+^ due to higher capacity of Cu^2+^ to nonspecifically bind biomolecules, particularly through thiol groups [33], (ii) using several mechanisms of reaction, Cu^2+^ can promote Fe^2+^-catalyzed Fenton reactions [34], (iii) unlike the system Fe^3+^ + H_2_O_2_, the system Cu^2+^ + H_2_O_2_ is able to produce large quantities of *·OH* using the following series of reactions [35]: 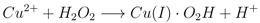
(3)

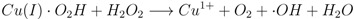
(4)

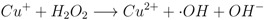
(5)

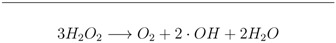
(6)

With the existence of so many deleterious O_2_-dependent mechanisms, it is not a surprise that, from the evolutionary point of view, the sudden release of large quantities of O_2_ to the environment has been considered an ecological disaster for primordial anaerobic life [25]. However, it is reasonable to speculate that the formation of banded iron (and the decrease of soluble Fe^2+^) during the first GOE could have at least contributed to diminish the Fe-associated Fenton damages, maybe even providing some time to develop survival strategies to tolerate the new pollutant. 

Interesting enough, when surveying for successful O_2_-protection mechanisms, we found that in most cases, the problem was solved by directly controlling the metal ions instead of the O_2_ pollutant. A few representative examples will be reviewed in the next sections. 

### 4.2. Handling Intracellular Labile Ion Pools

As stated in Section 3 and Section 4.1, although both Fe and Cu are transition metals, the water solubilities and O_2_ related reactivities of different redox species greatly determine the way these metals affect biomolecules. Consequently, different evolutionary strategies have been used to fine-tune Fe and Cu cellular homeostasis. A clear example of these differential strategies can be found in the way free Cu and Fe (labile ion pools) are controlled within the cells. 

**Figure 1:**
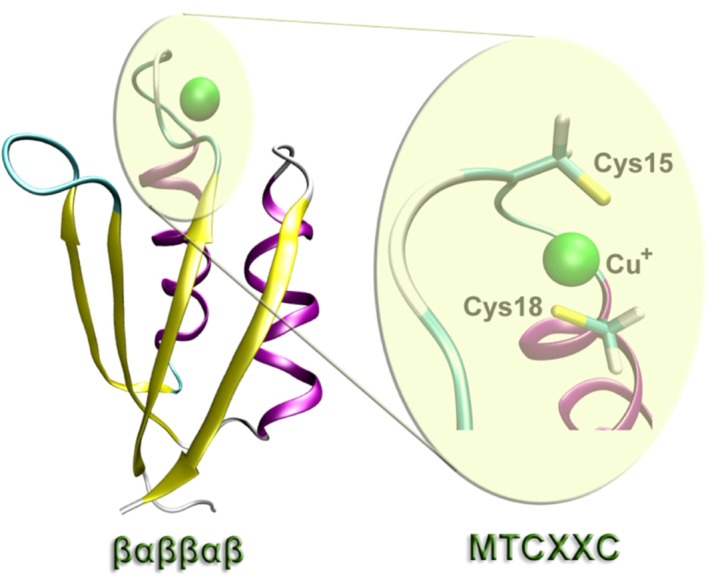
The Heavy Metal Associated (HMA) domain used to transport Cu ions [40]. This domain features the *βαββαβ* ferredoxin-like fold [41] featuring the sequence MTCXXC with two well conserved cysteines to bind Cu [42]. The figure specifically illustrates the case of Atx1 from *S. cerevisiae* in which the two cysteine residues are Cys15 and Cys18.

Intracellular copper ions (Cu^+/2+^) not bound to proteins are toxic [36] and as we already described in the previous section. It has even a higher toxic potential than Fe, and therefore in all living species intracellular Cu is tightly regulated. The intracellular concentration of Cu ions are kept close to zero due to the presence of a highly Cu-chelating environment in the cytosol [37]. In fact, using Cu fluorescent sensors, it has only been possible to find kinetically labile Cu pool in the mitochondria and golgi apparatus [38]. When there is a need of Cu, these ions are always transported using Cu chaperons proteins featuring a highly conserved Cu-binding domain (Figure 1) that is also present in other membrane Cu transport proteins like Ccc2 in *S. cerevisiae* or ATP7A and ATP7B in humans [39]. 

On the other hand, recently reviewed evidence [43] clearly indicates that two main sources contribute to the equilibrium between free Fe^2+^ and Fe^3+^ in the cytosol: (i) Fe exchange with the extracellular environment through the cell membrane, and (ii) Fe exchange with intracellular sources such as labile Fe pools in organelles. 

There are several other differences between Cu and Fe regarding transport and secretion from the cytosol. Cu expulsion is conducted through a conserved secretory pathway that is fundamentally identical between yeast and humans [44]. In this pathway, Cu is always bound to proteins that share the same basic domain (see Figure 1). Fe can also be transported by proteins in the cytosol, but opposite to case of Cu, no dedicated Fe chaperon has been identified. Instead, from yeast to humans, Fe can be intracellularly transported through bi-functional proteins that also participate in RNA binding [45,46]. Besides this, free Fe can also be secreted directly from the cytosol using cell membrane exporter proteins like ferroportin [43]. 

### 4.3. Intracellular Metal Storage

If metal ions are available in the cell, the inevitable consequence is the increase of Fenton chemistry. Therefore, a primary control mechanism involves “sequestering” free metal ions with metal binding proteins. Considering the relevant role of Fe^2+^ since the start of life, it is not surprising that Fe binding is a widespread protein function. Probably the best study Fe storage protein is ferritin that has evolved from simpler rubrerythrin-like molecules into a variety of isoforms that are expressed in most Archaea, Bacteria, and Eubacteria [47]. All along their evolutionary history, ferritins have function not only as pure Fe storage proteins; simultaneously they have been involved in free radical detoxification through their ferroxidase activity resumed in the following chemical reactions [48]:

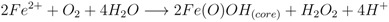
(7)

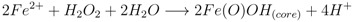
(8)

**Figure 2:**
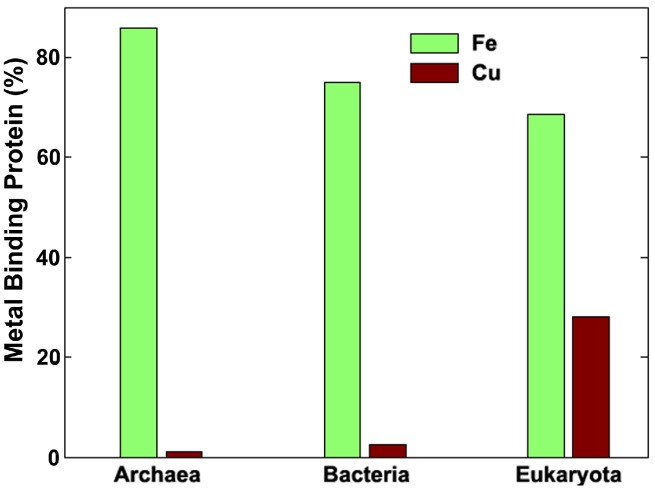
Distribution of Fe and Cu binding proteins per taxa. Note the small percent of Cu-binding proteins in Archaea with respect to Eukaryota and the inverted trend of this behavior for the case of Fe-binding proteins. Raw data for analysis came from Uniprot [30].

The driving force behind these reactions is the insolubility of the Fe^3+^ compounds, but this time instead of form banded iron minerals, Fe^3+^ goes through an alternative mineralization step forming a Fe core in the cavity of ferritin. On the other hand, Cu storage proteins seems to be relatively recent and less specialized than their Fe counterparts; a representative example is the simpler and unspecific metallothioneins (MTs) [49], which are absent in Archaea. They are present in prokaryotes, but their main occurrences are localized in eukaryotic cells [50]. Structurally, MTs are composed of no more than 60–70 amino acids, and around 30% of them are cysteines. However, the high variability in the size and the sequence of these group of proteins prevent them from been too specific regarding the metal binding capabilities, and besides Cu, they can bind zinc (Zn) [51] or cadmium (Cd) [52] but notably not Fe [53]. To illustrate this concept, we show in Figure 2 a plot of the percentages of Fe vs. Cu binding proteins within the three main domains of life found in the Uniprot database [30]. The data seems to indicate that the Cu binding problem is a relatively recent one in evolutionary biology and could probably be associated to the rise of oxygen in the planet. 

### 4.4. Control of the Oxidative Stress

Another way of protecting life from the effect of the Reactive Oxygen Species (ROS) produced by Fenton chemistry is the creation of specialized antioxidant mechanism: e.g., catalases and peroxidases for the elimination of peroxide (H_2_O_2_); superoxide-dismutases (SODs) for the elimination of the superoxide (*·O*_2_− ). The actions of these enzymes occur through the following general reactions:

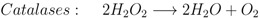
(9)

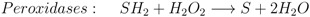
(10)

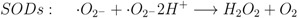
(11)

**Figure 3:**
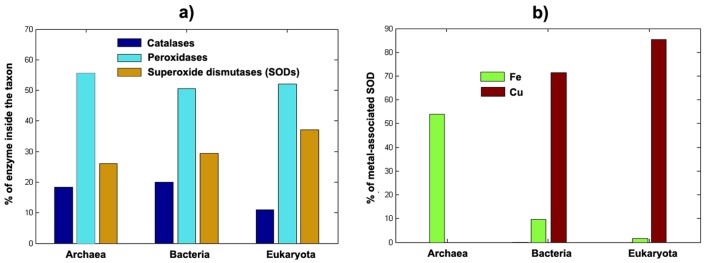
(a) Distribution oxidative stress enzymes per taxa. (b) Distribution of Fe and Cu SODs per taxa. Raw data for analysis came from Uniprot [30].

The utilization of these types of enzymes per taxa is presented in Figure 3a. While the distribution of peroxidases seems to be similar in the three taxonomic domains, two opposite tendencies are observed for the other antioxidant enzymes: (i) the catalases (which are mainly Fe-containing enzymes) are prevalently used in ancient cell types like those of Archaea and Bacteria, and (ii) from Archaea to the relatively modern Eukaryota there is an increase in the distribution of SODs, which are enzymes that use more Cu and less Fe as we move from Archaea to Eukaryota (see Figure 3b). In fact, it is particularly significant to note that while the Archaea group does not use Cu-containing SODs, Bacteria and Eukaryota use increasing quantities of Cu-containing SODs and decreasing quantities of Fe-containing SODs (Figure 3b). Although interesting, we should not hurry in extracting strong conclusions from these observations. However, it is not difficult to see a tendency in the modern and prevalently O_2_-exposed cell types (Eukaryota) to increasingly use antioxidant enzymes containing the more bioavailable Cu (SODs) and use less antioxidant enzymes that contain the less bioavailable Fe (catalases). 

### 4.5. Fe-and Cu-Mediated O_2_ Transport Proteins.

**Figure 4:**
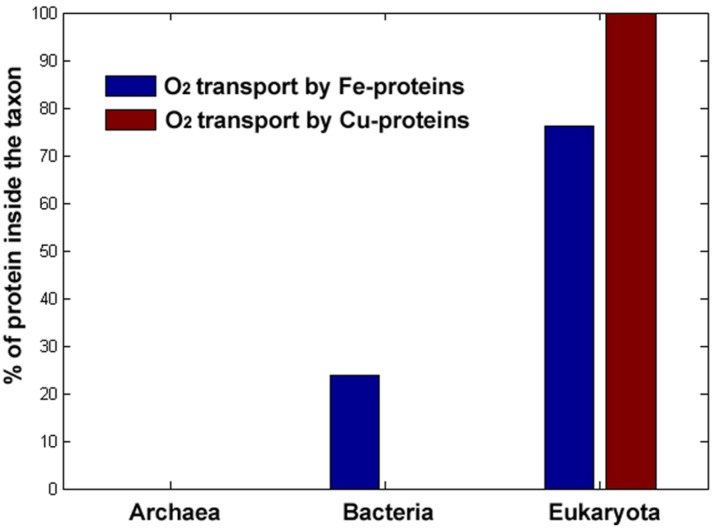
Distribution of Fe and Cu O_2_-binding proteins per taxa. Raw data for analysis came from Uniprot [30].

Given the high reactivity of O_2_ and its propensity for Fenton Chemistry using Cu and Fe (see section 4.1), an absolute absence of these metals in O_2_ transport proteins would be expected. However, as we show in Figure 4, this is not the case, and several transport proteins of this type exist in nature. 

As expected, in Figure 4 we see no evidence of this sophisticated O_2_ transport mechanism in the relatively ancient Archaean cell types, which evolved mostly in O_2_ deprived environments. Once again, we find the beginning of this mechanism in the versatile Bacteria domain, although employing only Fe-proteins. However it is in mostly O_2_-exposed Eukaryotes where this mechanism reaches sophistication and widespread distribution. Prototypical examples are the structure and mechanism of the Fe-containing hemoglobin in higher mammals [54] and the even rarer case of O_2_ transporting Cu-proteins that exist only in the Eukaryote domain, the prototypical example being the haemocyanins present in arthropods and mollusca [55]. 

## 5. Concluding Remarks

Life is full of surprises. Even today, biologists are amazed that even at the geologically violent beginnings of our planet it was still possible for life to be created from very modest geochemical principles. In spite of the early challenges to transform a group of geochemical processes in auto-reproducible biochemistry, new geological obstacles were faced by primitive living organisms. One of this obstacles, the rising of O_2_ in the primitive atmosphere, would in fact change the metal ion bioavailability and force the existing organisms to evolve adaptation of metals homeostasis either to avoid the danger associated with the excess of newly produced O_2_ or to even manipulate and use O_2_ in new ways. In this context, the study of the physical and chemical properties of Fe and Cu ions and the determination of their associated metabolic reactions in different organisms help us to improve our understanding of early evolution. The data in the literature seem to indicate that under the new atmospheric conditions, the Fe-based metabolism indeed decreased to give rise to a combination of Fe and Cu-related mechanisms. 
